# Semaphorin-6A controls guidance of corticospinal tract axons at multiple choice points

**DOI:** 10.1186/1749-8104-3-34

**Published:** 2008-12-08

**Authors:** Annette E Rünker, Graham E Little, Fumikazu Suto, Hajime Fujisawa, Kevin J Mitchell

**Affiliations:** 1 Smurfit Institute of Genetics, Trinity College Dublin, Dublin 2, Ireland; 2 Division of Developmental Genetics, National Institute of Genetics, Mishima 411-8540, Japan; 3 The 21st Century COE Program, Division of Biological Science, Nagoya University Graduate School of Science, Chikusa-ku, Nagoya 464-8602, Japan; 4 Center for Regenerative Therapies Dresden, Technical University of Dresden, 01307 Dresden, Germany

## Abstract

**Background:**

The trajectory of corticospinal tract (CST) axons from cortex to spinal cord involves a succession of choice points, each of which is controlled by multiple guidance molecules. To assess the involvement of transmembrane semaphorins and their plexin receptors in the guidance of CST axons, we have examined this tract in mutants of *Semaphorin-6A *(*Sema6A*), *Plexin-A2 *(*PlxnA2*) and *Plexin-A4 *(*PlxnA4*).

**Results:**

We describe defects in CST guidance in *Sema6A *mutants at choice points at the mid-hindbrain boundary (MHB) and in navigation through the pons that dramatically affect how many axons arrive to the hindbrain and spinal cord and result in hypoplasia of the CST. We also observe defects in guidance within the hindbrain where a proportion of axons aberrantly adopt a ventrolateral position and fail to decussate. This function in the hindbrain seems to be mediated by the known Sema6A receptor PlxnA4, which is expressed by CST axons. Guidance at the MHB, however, appears independent of this and of the other known receptor, PlxnA2, and may depend instead on Sema6A expression on CST axons themselves at embryonic stages.

**Conclusion:**

These data identify Sema6A as a major contributor to the guidance of CST axons at multiple choice points. They highlight the active control of guidance at the MHB and also implicate the inferior olive as an important structure in the guidance of CST axons within the hindbrain. They also suggest that Sema6A, which is strongly expressed by oligodendrocytes, may affect CST regeneration in adults.

## Background

**Figure 1:**
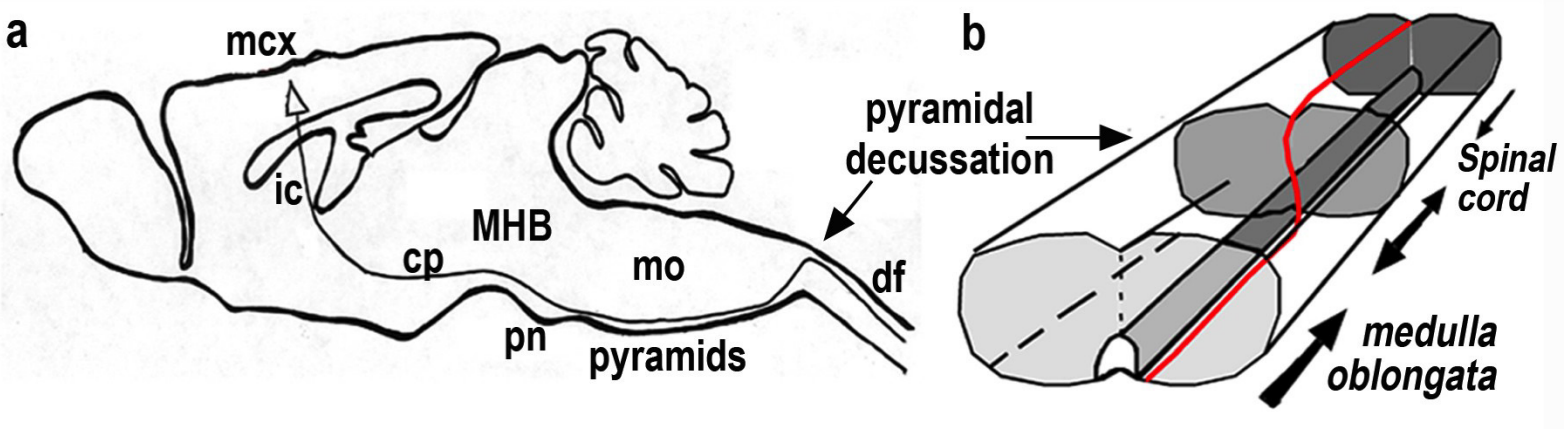
Schematic of the corticospinal tract (CST) trajectory. The course of the CST is shown in **(a) **a sagittal view of the mouse brain (black line) and in **(b) **a three-dimensional schematic of the medulla (red line). (a) The CST begins in the motor cortex (mcx), where layer V neurons project axons through the internal capsule (ic) and cerebral peduncles (cp) to the level of the mid-hindbrain boundary (MHB), just rostral to the pontine nuclei (pn), where they turn medially and ventrally to project along the ventral surface of the medulla oblongata (mo) as the pyramidal tracts. Within the caudal medulla the CST is surrounded laterally and dorsally by the inferior olives (not shown but visible in Figures 2d and 6). (b) At the boundary between the medulla and the spinal cord CST axons turn dorsally and cross the midline, forming the pyramidal decussation and subsequently project caudally into the dorsal funiculus (df).

The corticospinal tract (CST) is a well-defined model system for several neurodevelopmental processes, such as axon guidance, topographic connectivity, collateral sprouting and stereotyped pruning, which establish precise patterns of connectivity in the vertebrate nervous system [1]. The trajectory of the CST from the cortex to the spinal cord involves a succession of choice points (Figure 1), each of which is controlled independently, often by different sets of molecules (comprehensively reviewed in [1]).

Genetic analyses have revealed a number of specific choice points that are particularly vulnerable to genetic lesions, most probably because they involve sudden deviations in trajectory, crossing borders between embryonic compartments or departure from larger nerve pathways. For example, defects in the guidance of corticofugal projections, including CST axons, have been observed in various mutants in the initial projections from the cortex across the pallial-subpallial boundary (*Celsr3 *[2,3], *Frizzled-3 *[4,5], *Pax6 *[6]), within the internal capsule (*Ctip2 *[7]) and across the telencephalic-diencephalic border into the cerebral peduncles (*Nkx2-1 *[8], *Slits *[9], *Robos *[10]). *Ctip2 *mutants also display defects in CST projections more caudally, at the level of the pons [7]. A number of other mutants cause defects in CST projections in the hindbrain, especially at the junction of the medulla and spinal cord, where they form the pyramidal decussation. At this point, CST axons, which have been extending caudally in a ventral position near the midline, project dorsally and across the midline (forming an X, hence 'decus', from the Roman numeral) to join the dorsal funiculus, where they resume their caudal course. A variety of defects in CST projections at this region are observed in mutants of *L1CAM *[11-13], *EphA4 *[14-18], *NCAM *[19], and *netrin-1 *or netrin receptor genes [20]. Such defects are often, though not always, associated with hypoplasia of the CST, presumably due to secondary degeneration of mistargeted axons.

Understanding the molecular control of CST guidance is of clinical importance in two ways. First, a number of hereditary neurological disorders involve aberrant development and/or progressive degeneration of the CST, which generally results in spastic paraplegia [21,22]. For example, in L1 syndrome, caused by mutations in *L1CAM *[23], spasticity is due to CST hypoplasia, which is probably a result of a CST guidance defect at the pyramidal decussation, as seen in L1-deficient mice [11-13,24]. Joubert syndrome, which can be caused by mutations in five known genes [25,26], is also characterised by a failure of CST axons to decussate normally [27-31]. In addition, a recent study suggests that CST development may be compromised in adolescent-onset schizophrenia, which commonly involves motor symptoms [32]. Second, the elucidation of the developmental programme controlling CST axon guidance is highly relevant to the development of strategies to promote regeneration of spinal nerves following injury or degeneration [33]. Thus, guidance molecules with known functions in CST development have later been reported to improve CST regeneration after their manipulation in animal models for spinal cord injury, including L1 [34-36] and EphA4 [37].

Semaphorins have been implicated in the control of multiple aspects of neural development, including cell migration, axon guidance, dendritogenesis and many others [38,39]. Though secreted class 3 semaphorins have been better studied, transmembrane semaphorins, especially of class 6, have attracted recent interest with the discoveries that they are important mediators *in vivo *of axon guidance and cell migration in many different parts of the brain [18,40-44]. These functions seem to be mediated by direct and specific binding to members of the PlxnA subfamily [40,41,45,46]. There is good evidence that these transmembrane semaphorins and their invertebrate orthologues can also function as receptors [47-51]. Analyses of *Sema6A *mutants have highlighted the important role of this molecule in cell migration and axon guidance in many parts of the nervous system [18,42,44,45] and the complicated, context-dependent nature of its interactions with PlxnA2 and PlxnA4 [42,43,45,52].

Here, we identify defects in CST axon guidance in *Sema6A *mutants at the mid-hindbrain boundary (MHB) and in navigation over the pontine nuclei that dramatically affect how many axons arrive to the hindbrain and spinal cord. We also observe defects in the caudal medulla where many axons spread laterally, resulting in a failure to decussate. The latter role seems to be mediated by the known Sema6A receptor PlxnA4, but the former appears independent of this and of the other known receptor, PlxnA2, and may depend on early expression of Sema6A on CST axons themselves. These data identify Sema6A as a major contributor to the guidance of CST axons at multiple choice points.

## Results

### Hypoplasia and misrouting of the corticospinal tract in adult *Sema6A *mutants

**Figure 2:**
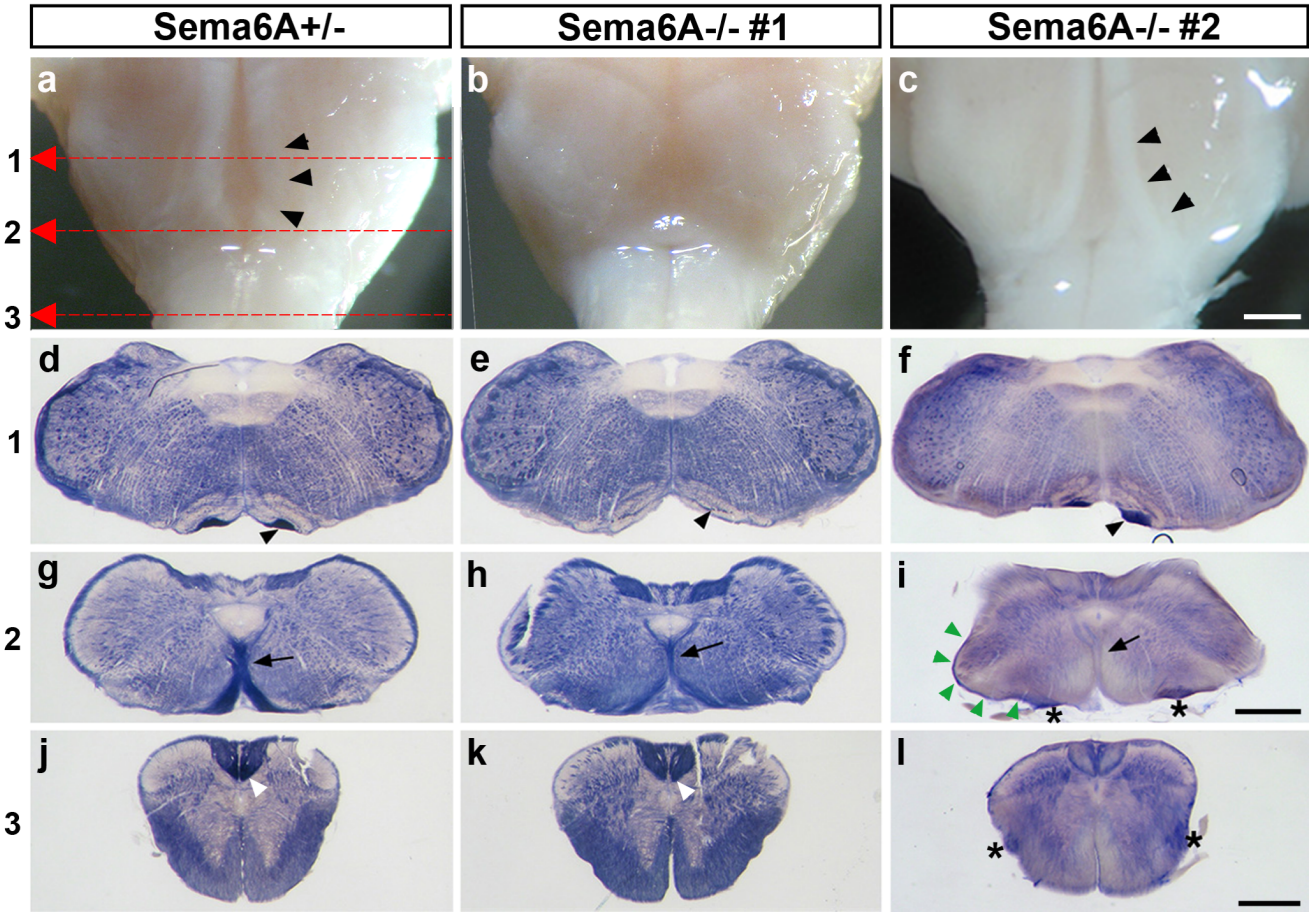
Moderate to severe hypoplasia and misrouting of the corticospinal tract (CST) in adult *Sema6A *mutants. **(a-c) **Macroscopic view of the ventral medulla in adult *Sema6A*^+/- ^and *Sema6A*^-/- ^animals. The pyramidal tracts are visible as heavily myelinated structures on either side of the midline (black arrowheads in (a, c)). Red arrows show levels of sections in (d-l): 1 (d-f); 2 (g-i); 3 (j-l). **(d-l) **PLAP-stained cross-sections from the same brains at indicated levels. In the *Sema6A*^+/- ^brain (a, d, g, j), the pyramidal tracts run close to the midline at the ventral surface of the medulla (black arrowhead in (a, d)) and disappear from macroscopic view at the level of the decussation, where they turn dorsally (black arrow in (g)) to enter the dorsal funiculus (white arrowhead in (j)). The middle column (b, e, h, k) shows an example of a *Sema6A *mutant with severe hypoplasia of the CST. In these severe cases the CST is not visible as the medullar pyramids macroscopically (b). Within the medulla the few remaining axons do not run at the surface but deeper, embedded within the inferior olive (black arrowhead in (e)). These fibres mostly decussate correctly (black arrow in (h)) and enter the dorsal funiculus of the spinal cord (white arrowhead in (k)). The right column (c, f, i, l) shows another mutant where the size of the pyramids is reduced to different degrees on either side, but remains visible macroscopically as a tight bundle at the surface (black arrowheads in (c, f)). At the caudal olive the majority of axons track away from the midline and are midway towards the lateral border at the level of the decussation (black arrowheads in (c); asterisks, green arrowheads in (i)). Here they do not decussate but proceed down the spinal cord in a ventrolateral position (asterisks in (l)). A small portion of CST axons that remain close to the midline up to the level of decussation cross contralaterally and dorsally (black arrow in (i)). Scale bars 500 μm: the bar in (c) is for (a-c); the bar in (i) is for (d-i); the bar in (l) is for (j-l).

Mutants in *Sema6A *were generated in a gene trap screen and have been previously described [18,53]. Macroscopic examination of the hindbrains of adult *Sema6A *mutants (n = 9) in comparison with wild-type (n = 3) and *Sema6A *heterozygous mice (n = 12) revealed dramatic defects in the size and trajectory of the CST. This tract was visible in wild-type and *Sema6A*^+/- ^mice as a highly myelinated tract running at the ventral surface of the medulla (Figure 2a). In contrast, in many *Sema6A *mutants (four out of nine) the CST was not visible as the medullar pyramids macroscopically (Figure 2b). In other mutants (five out of nine), the pyramids remained visible macroscopically, that is, they appeared as a tight bundle at the surface, but their size was reduced to various degrees between animals as well as between pyramids on either side of individuals (Figure 2c).

We used staining for the reporter gene placental alkaline phosphatase (PLAP), which is encoded on the gene trap cassette inserted into the *Sema6A *locus [18], to examine the CST of adult *Sema6A*^+/- ^and *Sema6A*^-/- ^mice at a microscopic level (Figure 2d–l). In adults, Sema6A, and thus PLAP, is strongly expressed in oligodendrocytes. Therefore, staining for PLAP labels all myelinated fibres in adults with one or both mutant alleles. The stainings confirmed a wide range in severity of CST size reduction and showed a reasonably smooth distribution in severity across the mutants analysed, including those that had no macroscopically visible CST. In addition, PLAP stainings revealed defects of the remaining CST axons. Instead of running at the surface of the medulla as a tightly fasciculated bundle as seen in *Sema6A*^+/- ^mice (Figure 2d, g, j), they were often split into several bundles that ran either normally at the surface (Figure 2f) or aberrantly deeper within the medulla (that is, at the rostrocaudal level of the inferior olive, they are embedded within the olive; Figure 2e, arrowhead). More strikingly, at the caudal olive a variable proportion of the remaining CST axons projected away from the midline towards the ventrolateral spinal cord (Figure 2i; but also detectable macroscopically when the CST remained visible at the surface, Figure 2c, arrowheads). Such axons failed to decussate and instead proceeded down the spinal cord in a ventrolateral position (Figure 2l, asterisk). The small portion of CST axons that remained close to the midline decussated correctly and entered the dorsal funiculus of the spinal cord (Figure 2h, k).

### Corticospinal tract guidance defects at earlier choice points

The hypoplasia of the CST might be the result of a guidance defect at the pyramidal decussation with many axons dying because they do not find their targets, while others manage to contact their targets even after misguidance and via an alternative route. Another possibility is that hypoplasia of the tract at the level of the medulla is due to fewer axons reaching that point in the first place; that is, that there is an additional choice point along the CST trajectory at which axons are misguided and fail to proceed down the spinal cord. To test these ideas we traced the CST during its development, that is, at postnatal day (P)4, when the majority of CST axons have just arrived in the spinal cord.

**Figure 3:**
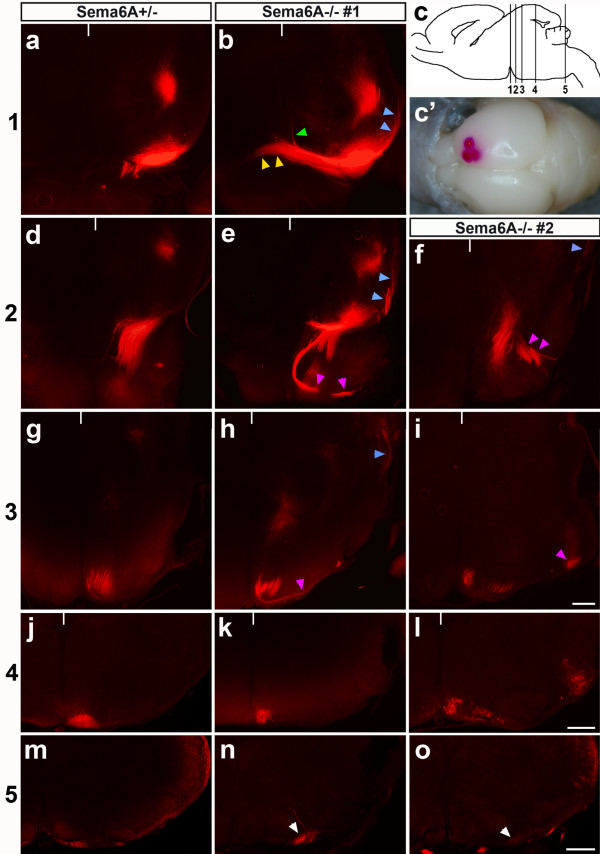
*Sema6A *mutants have corticospinal tract (CST) axon guidance defects at the mid-hindbrain boundary (MHB) and in the caudal medulla. **(a-o) **Postmortem DiI placement into the motor cortex of P4 *Sema6A*^+/- ^(left column) and *Sema6A*^-/- ^(middle and right columns) brains (example in (c')). Levels of shown cross-sections are indicated on a schematic sagittal brain section shown in (c): 1 (a, b); 2 (d-f); 3 (g-i); 4 (j-l); 5 (m-o); white notches indicate the position of the midline in (a, b, d-l). At the MHB of *Sema6A*^+/- ^mice, traced fibres at the ventrolateral surface turn and travel halfway towards the midline (a), then turn ventrally and project over the pontine nuclei (d) to finally reach the ventral surface of the medulla (g) to form the pyramids (j, m). At the MHB of *Sema6A*^-/- ^mutant mice, labelled fibres took several aberrant routes: a dorsal turn at the caudal end of the cerebral peduncle (blue arrowheads in (b, e, h, f)), midline crossing (large fraction; yellow arrowheads in (b)) or dorsal turning after a correct turn towards the midline (minor fraction; green arrowhead in (b)), or, after correct reorientation ventrally, splitting into several bundles, of which some project through the pontine nuclei and/or laterally (magenta arrowheads in (e, h) or (f, i), respectively). In the medulla of *Sema6A*^+/- ^mice, traced fibres travelled close to the midline (j). This could also be observed for the reduced number of labelled axons that were routed properly at the MHB of *Sema6A *mutants (k, l). However, at the level of the inferior olive some (o) or all (n) of these axons projected away from the midline and proceeded ipsilaterally at a ventrolateral position (white arrowheads in (n, o)), in contrast to those in *Sema6A*^+/- ^mice, which remained close to the midline (m) and subsequently decussated. Scale bars 200 μm: the bar in (i) is for (a, b, d-i); the bar in (l) is for (j-l); the bar in (o) is for (m-o).

Placement of small crystals of the lipophilic dye DiI into the motor cortex of P4 post-mortem brains effectively labelled the descending CST (Figure 3). In *Sema6A*^+/- ^mice (n = 4; Figure 3, left column), traced fibres could be followed through the internal capsule and cerebral peduncle, revealing the normal trajectory of these fibres through the brain. At the MHB, traced fibres extending longitudinally on the ventrolateral surface of the midbrain turn medially and travel halfway towards the midline, then turn ventrally over the pontine nucleus to finally reach the ventral surface of the medulla to form the pyramids.

In Sema6A mutants (n = 5) we did not observe major trajectory defects of traced axons rostral to the MHB and the gross amount of traced fibres immediately rostral to the MHB was comparable to that seen in Sem6A heterozygous mice (data not shown; Figure 3a, b). However, at the MHB of *Sema6A *mutant mice (Figure 3, middle and right columns) we observed traced fibres taking several aberrant routes. At the caudal midbrain, most axons initially made a correct medial turn but some turned dorsally instead (three of five; Figure 3b, e, f, blue arrowheads). Of the axons that turned medially, a large fraction failed to turn ventrally over the pontine nuclei and instead projected all the way to the midline (five of five) and even crossed it (four of five), below the interpeduncular nucleus (four of five), or, as a minor fraction, turned dorsally (three of five; Figure 3b, yellow or green arrowheads, respectively) or ventrally (two of five). In the ventral projection over the pontine nuclei the tract appeared split into a couple of bundles. Some axons ran correctly close to the midline but others could be found to split from this main bundle to take an aberrant route within the pons or towards lateral areas (three of five; Figure 3e, f, h, i, pink arrowheads).

Further caudally, in *Sema6A*^+/- ^mice, traced fibres travelled through the medulla close to the midline (Figure 3j). This could also be observed for the reduced amount (to varying degrees) of axons that were routed properly at the MHB of *Sema6A *mutants (Figure 3k, l). However, at the level of the inferior olive, which appeared to form normally in *Sema6A *mutants, some (two of four) or all (two of four) of these axons projected away from the midline and were some distance away from the spot where CST axons of wild-type mice would turn dorsally and cross the midline (Figure 3n, o). These axons proceeded ipsilaterally at a ventrolateral position.

### Expression of *Sema6A *and its interacting partners

Sema6A is known to mediate some effects through interactions with PlxnA2 [42,52] and/or PlxnA4 [40,45]. Some members of this class of transmembrane semaphorins or their orthologues in invertebrates have also been shown to be capable of reverse signalling, presumably as receptors [47-51]. To assess whether these plexin proteins might be involved in mediating these functions of Sema6A and/or whether Sema6A might be acting cell-autonomously in CST axons, we examined the expression patterns of these genes in motor cortex and in the regions of the midbrain and hindbrain where defects are observed.

**Figure 4:**
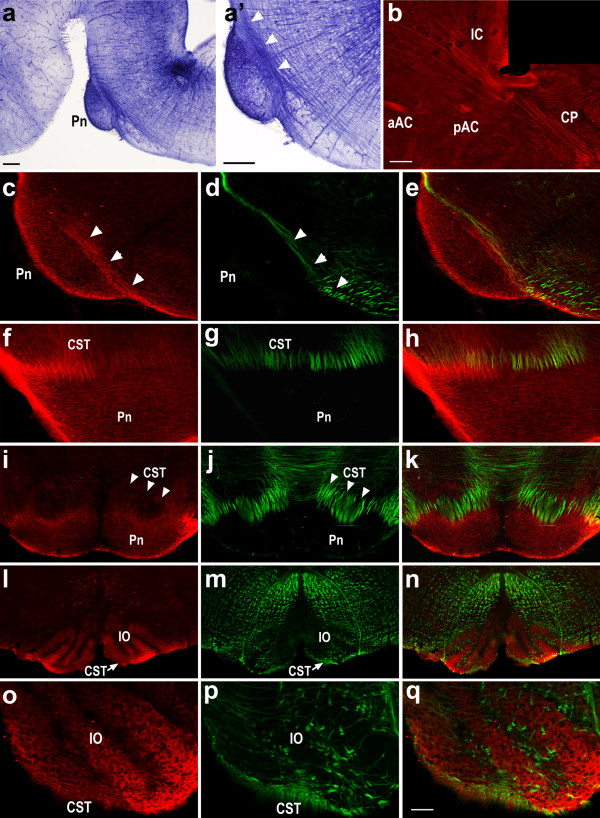
Expression of Sema6A protein at late embryonic stages. **(a, a') **Staining for the reporter PLAP on sagittal E17.5 sections from Sema6A^+/- ^mice. **(b-q) **Immunohistochemistry for Sema6A (red) and, as reference stain to visualize fibres, neurofilament M (green) on sagittal (b-e) (rostral is left) and coronal (f-q) sections of E17.5 (b-h) and P1 (i-q) wild-type mice. At E17.5, PLAP staining shows that Sema6A is expressed by corticospinal tract (CST) neurons (a, a'); arrowheads in a' show CST axons. At the same age, anti-Sema6A staining highlights corticofugal projections that travel through the internal capsule (ic) and cerebral peduncle (cp) (b), as well as CST axons as they pass the pontine nuclei (Pn; arrowheads in (c, d)) (c-h). Sema6A staining on CST axons at the level of the pons is no longer visible at P1 (i-k) (arrowheads in (i, j)) and is also lacking from these axons as the pass the inferior olive (IO). Along the CST trajectory, Sema6A immunoreactivity was detected at lower levels in the Pn (c, f, i) at E17.5 and P1, and at moderate to high levels in the IO at E17.5 (l, o). aAC, anterior arm of the anterior commissure; pAC posterior arm of the anterior commissure. Scale bar in (a, a', b) is 200 μm. Scale bar in (q) is: 100 μm for (c-h); 200 μm for (i-n); 50 μm for (o-q).

We used reporter gene staining, RNA *in situ *hybridization and immunohistochemistry to examine Sema6A expression, which we found to be highly dynamic. At embryonic day (E)17.5, CST axons have crossed the mid-hindbrain border and reached the pons. At this stage, CST axons stain positively for PLAP in Sema6A gene trap embryos (Figure 4a, a'). This is far prior to the onset of myelination, demonstrating that neurons that give rise to the CST normally express Sema6A in late embryogenesis. To examine the distribution of the Sema6A protein itself, we performed immunohistochemistry on sections from E17.5 and P1 brains, using a commercially available polyclonal antibody against murine Sema6A (Figure 4b–q), which we proved to be specific for Sema6A by comparing stainings on wild-type and Sema6A mutant brain sections (Additional file 1). At E17.5, we observed Sema6A-immunopositive corticofugal fibres at the level of the internal capsule and cerebral peduncle (Figure 4b), and positive CST axons at the level of the MHB and pons (Figure 2c). Sema6A was also expressed in the MHB region by the pontine nuclei (Figure 4c–h). By postnatal stage P1, localisation of Sema6A on CST axons was no longer evident, either at the MHB, where expression in the pons persists (Figure 4i–k), or in the medulla, where the inferior olive was strongly Sema6A-immunopositive (Figure 4l–q).

**Figure 5:**
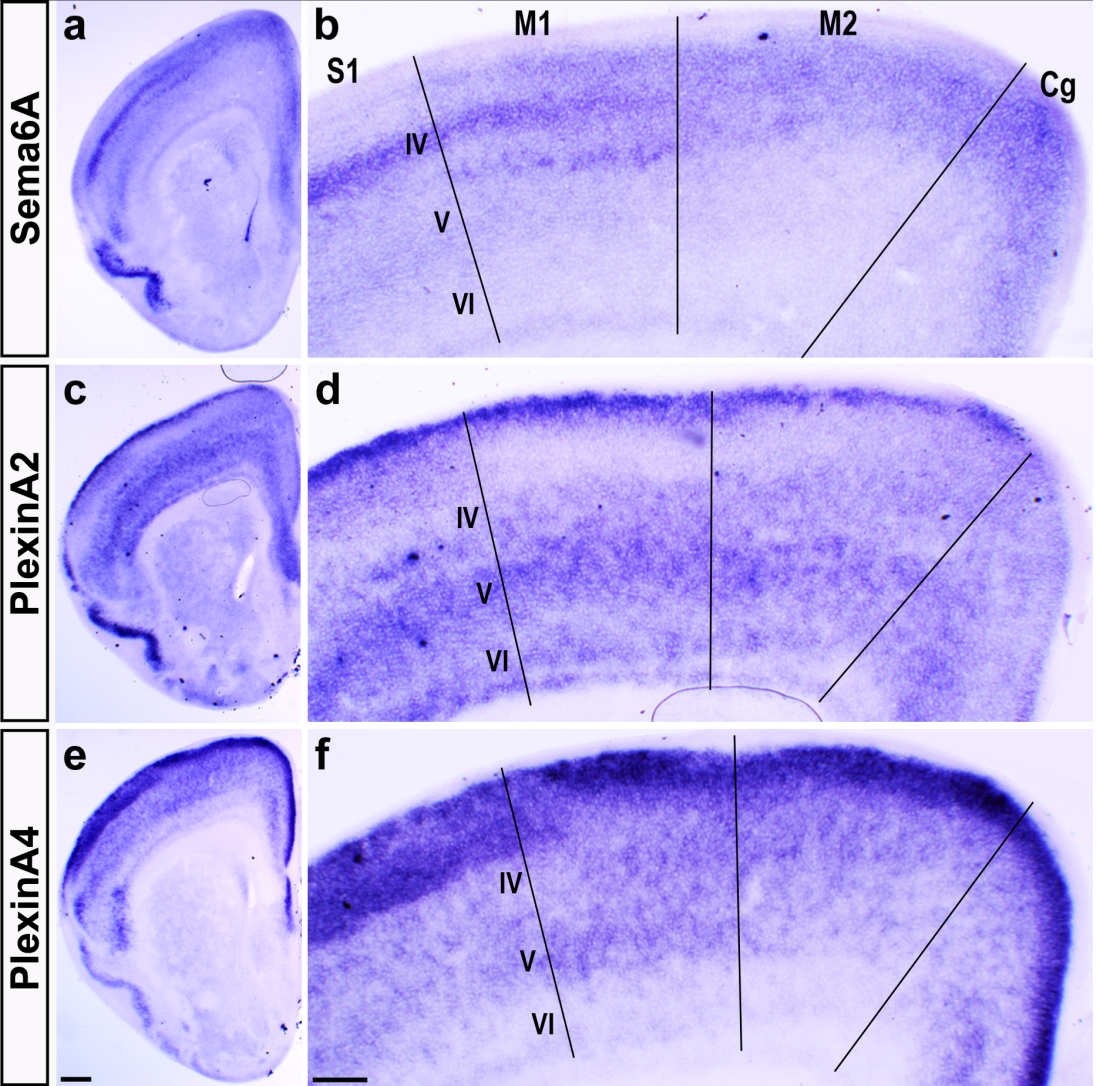
Expression of Sema6A, PlxnA2 and PlxnA4 in motor cortex. *In situ *hybridization at P4 reveals laminar and areal-specific expression of Sema6A, PlxnA2 and PlxnA4. **(a-f) **Rostral sections including the motor cortex are shown at low magnification (a, c, e) and at higher magnification (b, d, f). Within primary somatosensory cortex (S1), Sema6A is expressed strongly in layer IV (a, b), while PlxnA2 is expressed strongest in infragranular layers (but also in upper layers (c, d)) and PlxnA4 is expressed strongest in supragranular layers (e, f). In primary motor cortex (M1), Sema6A is expressed in layer IV and upper layer V. PlxnA2 and PlxnA4 are both expressed throughout layer V. PlxnA2 is also expressed in layers I, IV and VI of M1 and PlxnA4 in all layers except layer VI. M2, secondary motor cortex; Cg, cingulate cortex. Scale bar in (e) is 200 μm and is for (a, c, e); scale bar in (f) is 100 μm and is for (b, d, f).

We used *in situ *RNA hybridization to examine expression of *Sema6A*, *PlxnA2 *and *PlxnA4 *in the neocortex at postnatal stage P4. This revealed laminar and areal-specific staining for each of the three genes (Figure 5). The pattern of layer-specificity is complementary between *Sema6A *and the *Plxn *genes in some areas (for example, primary somatosensory cortex (S1)). The laminar patterns also change in an areal-specific manner, especially between S1 and primary motor cortex (M1). Within the primary motor cortex all three genes are expressed to some extent in layer V, the source of CST axons. Sema6A mRNA at these stages is expressed highly in upper layer V, PlxnA2 mRNA has the highest expression throughout layer V, and PlxnA4 mRNA is expressed moderately, also throughout the layer. Corticospinal projection neurons are found predominantly in deep layer V [54,55]. Consistent with this, and with the lack of antibody staining at this age, staining for PLAP activity in Sema6A heterozygotes does not strongly label CST axons at postnatal stages (data not shown). As PlxnA1 and PlxnA3 have also been indirectly implicated in Sema6A functions [43,56], though not shown to bind to it directly, we also examined the expression patterns of the genes encoding them in the motor cortex. While both genes are expressed in the cortex at this age, neither is particularly strongly expressed by deep layer V neurons in M1 (Additional file 2).

**Figure 6:**
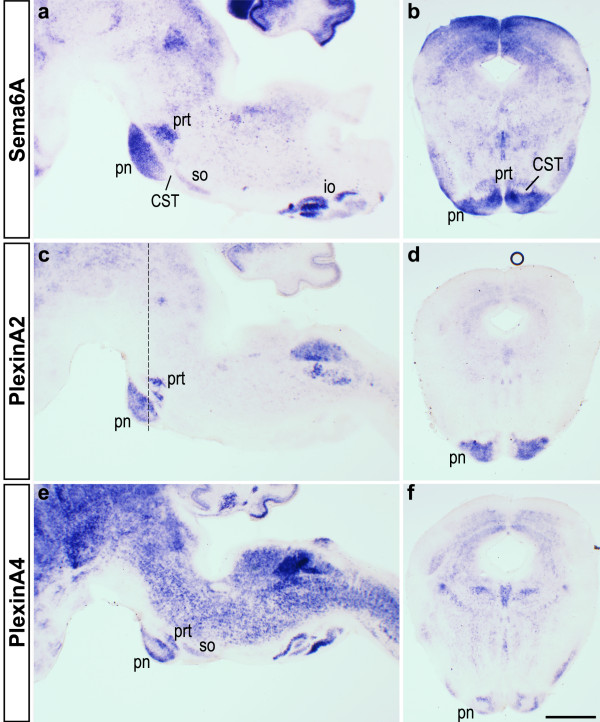
Expression of Sema6A, PlxnA2 and PlxnA4 in the hindbrain. **(a-f) ***In situ *hybridization for Sema6A, PlxnA2 and PlxnA4 at P0–P1, in sagittal (a, c, e) and coronal (b, d, f) planes. Strong expression of Sema6A (a, b) is observed in the pontine nuclei (pn) and other structures close to the corticospinal tract (CST) at the level of the pons, including the pontine reticular nucleus (prt) and superior olive (so), as well as in the inferior olive (io). PlxnA2 (c, d) and PlxnA4 (e, f) are expressed in distinct but overlapping patterns in these structures. In the inferior olive, PlxnA2 expression could not be detected at this age (Figure 6). The dashed line in (c) shows the approximate level of coronal sections. Scale bar: 500 μm.

**Figure 7:**
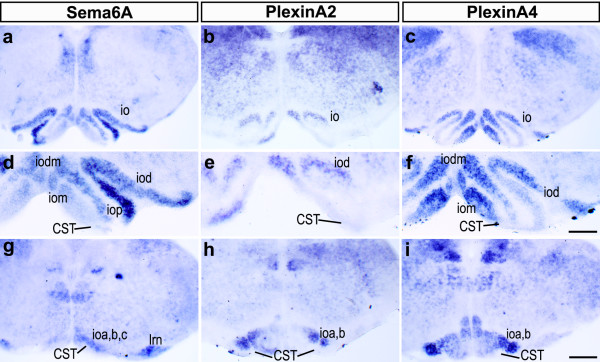
Expression of Sema6A, PlxnA2 and PlxnA4 in the caudal medulla. **(a-i) ***In situ *hybridization for Sema6A (a, d, g), PlxnA2 (b, e, h) and PlxnA4 (c, f, i) on coronal sections of the caudal medulla at P4. Sema6A and PlxnA4 are strongly expressed in the inferior olives in a partly complementary fashion (compare (a, d) with (c, f)). PlxnA2 is weakly expressed at this age in the inferior olives, in a pattern similar to PlxnA4 (b, e). Close to the level of decussation, all three molecules are expressed in the caudal portions of the inferior olive (io) (g-i). CST, corticospinal tract; ioa, b, c, subnuclei A, B, C of the medial nucleus of the inferior olive; iod, dorsal nucleus; iom, medial nucleus; iodm, dorsomedial cell group; iop, principal nucleus. Scale bar in (i) is 200 μm and is for (a-c, g-i); scale bar in (f) is 100 μm and is for (d-f).

At the level of the MHB, RNA *in situ *hybridisation confirmed expression of Sema6A by the pontine nuclei and also by the pontine reticular nuclei (Figure 6). The CST runs between these structures at this point. Just caudally, Sema6A is also expressed by the superior olivary nuclei. PlxnA2 and PlxnA4 are also expressed by the pontine nuclei, in distinct patterns, and by other structures in this region (Figure 6). Just rostral to the level of the pyramidal decussation, Sema6A and PlxnA4 are strongly expressed in the inferior olives at P4, in a partly complementary fashion (Figures 6 and 7). PlxnA2 is weakly expressed in this structure at this age, in a pattern that is very similar to that of PlxnA4. At this point, CST axons are anatomically constrained near the midline by the inferior olives.

As the expression data suggested the possible involvement of the *Plxn *genes, we investigated further whether similar CST guidance defects can be found in mutants for *PlxnA2 *and *PlxnA4*.

### Corticospinal tract guidance defects in *PlxnA4 *but not *PlxnA2 *mutants

**Figure 8:**
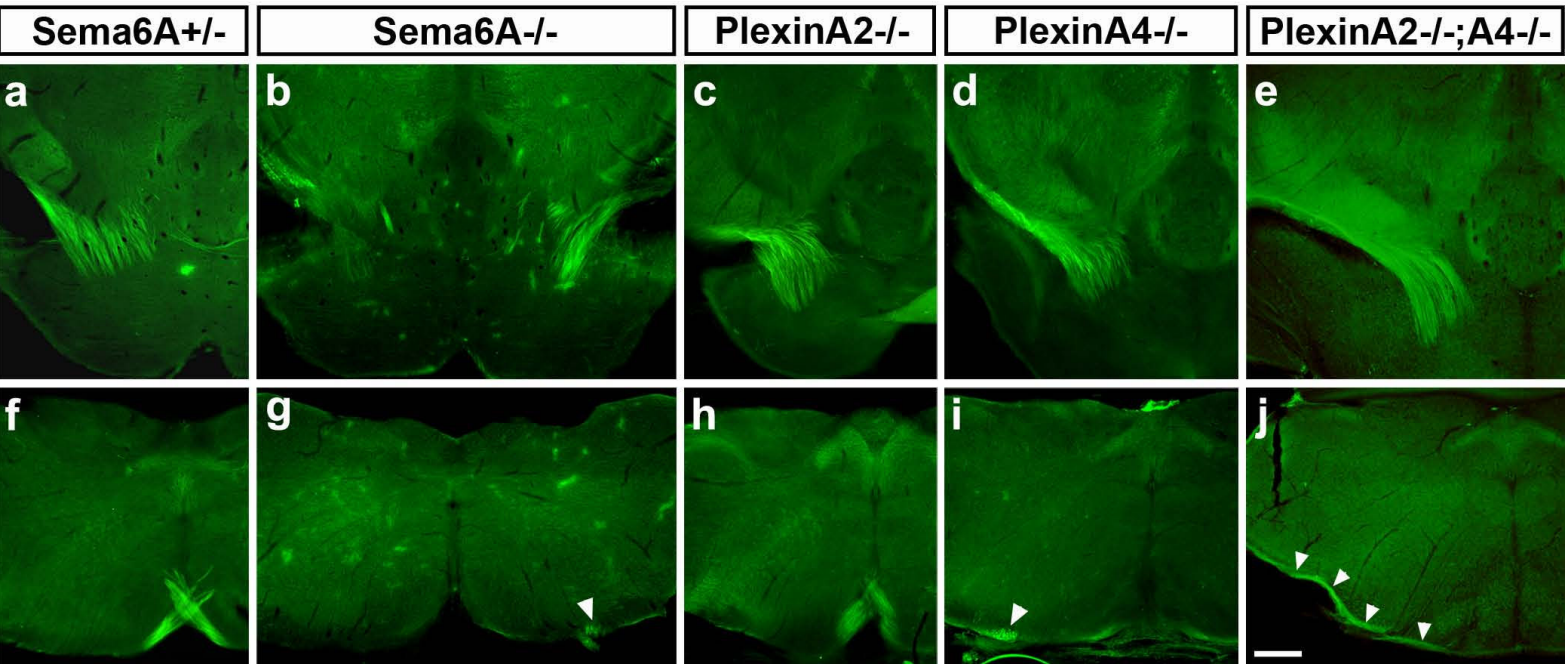
Corticospinal tract (CST) projections in *Sema6A *and *Plxn *mutants. **(a-j) **Anti-L1-immunohistochemistry on P10 *Sema6A*^+/- ^(a, f), *Sema6A*^-/- ^(b, g), *PlxnA2*^-/- ^(c, h), *PlxnA4*^-/- ^(d, i), and *PlxnA2*^-/-^*;PlxnA4*^-/- ^(e, j) mice. Anti-L1 staining in *Sema6A*^+/- ^mice highlights corticospinal tract (CST) axons turning ventrally over the pontine nuclei (a) and decussating at the caudal medulla (f). At the MHB of *Sema6A*^-/- ^mice (b), the number of CST axons is severely (left side) or moderately (right side) reduced. Only a few misguided axons can be found, indicating that most have already died between P4 and P10. At the decussation level (g), in some cases virtually no axons are left (left side). If CST axons are visible, they form a ventrolateral projection (arrowhead; right side), decussate properly (not shown; Figure 1), or split into two bundles to do both (not shown; Figure 1). *PlxnA2*^-/- ^mice show no apparent guidance defects or reduction in size of the CST (c, h). In *PlxnA4*^-/- ^mice, the size of the CST appears normal from the MHB (d), where no misguided axons can be found, up to the level of the caudal medulla. Here, the CST splits into two bundles that either form a ventral projection (white arrowhead in (i)) or join the contralateral dorsal funiculus (not shown). The phenotype in *PlxnA2;PlxnA4 *double mutants is indistinguishable from that in *PlxnA4*^-/- ^single mutants, with no apparent defect at the MHB (e) and an identical phenotype in the caudal medulla (j); white arrowheads indicate ventrolaterally projecting CST axons. Scale bar: 200 μm.

Immunohistochemistry with antibodies against the cell adhesion molecule L1 stains CST fibres selectively at P10. Using this method, we analysed the CST projections in *PlxnA2 *(n = 3), *PlxnA4 *(n = 4), and *PlxnA2;PlxnA4 *double (n = 2) mutants and compared them to *Sema6A *mutants (n = 2) or normal control mice (*Sema6A*^+/-^, n = 1; wild-type, n = 2; *PlxnA2*^+/-^, n = 1; *PlxnA4*^+/-^, n = 1) (Figure 8).

At the MHB of *Sema6A*^-/- ^mice at P10, the number of CST axons was already moderately to severely reduced. Only a few misguided axons could be found, indicating that the majority of such axons had already died between P4 and P10. At the decussation level, in some cases virtually no axons were visible. When present at this level, CST axons could be seen either forming a ventrolateral projection, decussating properly or split into two bundles to do both.

*PlxnA2*^-/- ^mice showed no apparent guidance defects or reduction in size of the CST. In *PlxnA4*^-/- ^mice, the size of the CST appeared normal from the MHB, where no misguided axons could be found, up to the level of the caudal medulla. Here, the CST split into two bundles that either formed a ventral or ventrolateral projection or decussated normally to join the contralateral dorsal funiculus. This defect, which is fully penetrant, thus strongly resembles that seen in *Sema6A *mutants.

Double mutants of *PlxnA2 *and *PlxnA4 *show very reduced viability and are thus extremely difficult to obtain. We have analysed two double mutants at P10, using anti-L1-immunohistochemistry. These showed no evidence of the defects observed at the level of the MHB and pons in *Sema6A *mutants (Figure 8). In particular, there was no apparent reduction in the number of fibres passing the pons. The defect in the caudal medulla was essentially the same as that observed in *PlxnA4 *single mutants. These data suggest that PlxnA2 is not involved in CST guidance in these regions and does not compensate for the lack of PlxnA4 at the MHB.

### *PlxnA4 *mutants do not show guidance defects at the mid-hindbrain boundary or corticospinal tract hypoplasia

**Figure 9:**
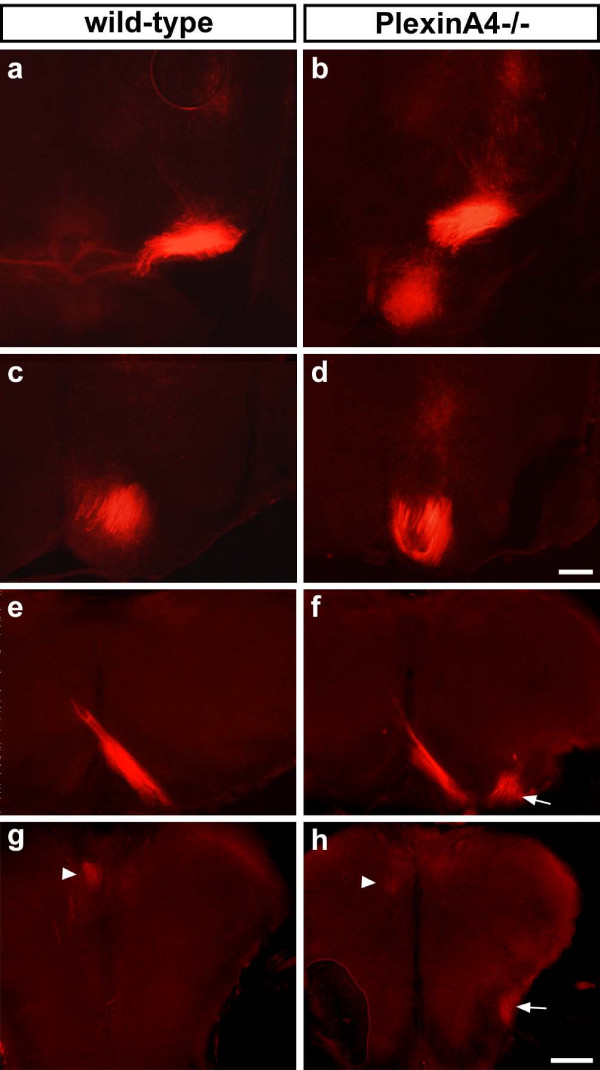
Corticospinal tract (CST) defect at the pyramidal decussation of *PlxnA4 *mutants. **(a-h) **Post-mortem unilateral DiI placement into the motor cortex of P4 wild-type (a, c, e, g) and *PlxnA4 *mutant brains (b, d, f, h). Traced CST axons in wild-type project past the pons and decussate normally (a, c, e) and enter the dorsal funiculus (arrowhead in (g)). Tracings at this age in *PlxnA4 *mutants show no apparent misguided axons at the MHB (b, d), but the same defect at the level of the decussation as described for the L1-staining at P10, with many ventrolaterally projecting axons (arrows in f, h) and a few correctly decussating axons (arrowhead in h). Scale bar: 200 μm.

To investigate the possibility that misguided axons were not detected at the MHB at P10 in *PlxnA4*-deficient mice because they might have already died, we traced the CST in these mutants at P4 (Figure 9). The tracing studies confirmed the CST defect at the pyramidal decussation (two of five animals showed no decussating axons, but only ventral projection; three of five mutants showed splitting into decussating and ventrally projecting axons; Figure 9) as described for the anti-L1-staining at P10. We did not find any traced fibres that were misguided at the MHB in *PlxnA4 *mutants (n = 5 animals, versus *PlxnA4*^+/-^, n = 5, and wild-type, n = 2; data not shown).

## Discussion

We describe two independent defects in CST guidance in *Sema6A *mutants, the first at the MHB, where many CST axons are misrouted and fail to reach the medulla, and the second in the caudal medulla, where many of the remaining axons adopt a ventrolateral position and fail to decussate. Although both PlxnA2 and PlxnA4 are expressed by neurons in deep layer V of motor cortex (the origin of the CST), *PlxnA2 *mutants do not show any defects in the CST. *PlxnA4 *mutants show the same phenotype as *Sema6A *mutants in the caudal medulla but neither *PlxnA4 *single nor *PlxnA2;PlxnA4 *double mutants show a defect at the MHB. Expression data suggest that the defect at the MHB may reflect a requirement for expression of Sema6A on CST axons themselves at early embryonic stages. They also implicate the inferior olives as an important source of guidance cues, which act to constrain the CST axons near the midline of the medulla where they can respond to cues for decussation. This model has implications for the interpretation of CST defects observed in other mutants, which we discuss below.

### Guidance at the mid-hindbrain boundary

The phenotypes observed in *Sema6A *mutants identify the MHB as a major choice point, or series of choice points, for CST axons. In fact, CST axons must perform rather complicated manoeuvres to successfully navigate this region and reach the hindbrain. First, exiting the cerebral peduncle at the caudal end of the midbrain, the CST turns sharply from a longitudinal direction towards the midline. Before reaching the midline, it must subsequently turn from this transverse direction ventrally to course over the pons. In parallel, the CST must partially defasciculate to allow collateral branching into the pons while retaining directional projection to reach and refasciculate at the ventral surface of the medulla and form the pyramids. Defects in each of these events can be observed in *Sema6A *mutants, thus identifying Sema6A as the first guidance molecule controlling CST trajectory at the MHB. Mutations in *Ctip2*, which encodes a transcription factor expressed in CST neurons, also result in defects at the MHB, where the tract is disorganised and defasciculated, with many axons failing to turn ventrally at the pons and no axons extending past the pons by P0 [7]. While these phenotypes reinforce the notion of this region as a complicated point in the trajectory of CST axons, they are somewhat distinct from those observed in *Sema6A *mutants.

We see a number of possible scenarios to explain the phenotypes observed. First, Sema6A could act directly in this region in canonical fashion as a repulsive ligand to constrain CST projections to an appropriate channel. This fits generally with the observation that Sema6A expression in this region seems to surround the CST. However, some of the phenotypes, such as midline crossing, are not obviously consistent with this model, given the absence of prominent midline expression of Sema6A. The apparent lack of involvement of the known Plxn receptors might also argue against it. Second, Sema6A could act as an attractive or permissive cue at this level. CST axons project in apposition to a number of pontine nuclei that are strongly Sema6A-positive. Changing responsiveness to guidance cues at different points of an axon's trajectory is a common mechanism [57], which has been observed directly with semaphorins [58-62]. The importance of Sema6A expression in pontine nuclei is, however, called into question by analyses of mutants in *Netrin-1 *or its receptor, *deleted in colorectal cancer *(*DCC*). In these mice, the pontine nuclei and pontine reticular nuclei fail to condense due to defects in cell migration [63]. While these mice show defects in CST guidance in the hindbrain (see below) they do not show any defects in CST guidance at the MHB. These findings suggest that the phenotype at the MHB in *Sema6A *mutants may instead be due to a lack of Sema6A expression on CST axons themselves during late embryogenesis. Sema6A could act in CST axons as a receptor for some unknown ligand in surrounding cells or, acting either as a ligand or a receptor (or both), could mediate axon-axon interactions that are important for proper guidance at this level. The orthologous *Drosophila *gene *Sema-1a *appears to function in such a fashion in motor axon guidance by setting an appropriate level of fasciculation between motor axons, allowing them to respond to guidance cues at specific choice points [64].

Our analyses at early postnatal stages indicate that the defects at this point are the most likely cause of the CST hypoplasia observed in adult *Sema6A *mutants. This is based on several observations: first, in none of the traced mutants did we see a gross difference in the number of CST axons arriving to the MHB at early postnatal stages. Although smaller defects within the forebrain might have been left undetected, we consider this possibility unlikely to be able to account for the significant hypoplasia observed. Second, many CST axons take widely divergent routes at this point and fail to enter the hindbrain. The failure to connect with appropriate targets is likely to result in the degeneration of these axons, which are not detected at later stages, and consequently the death of layer V cells themselves. Indeed, we do not observe any obvious derailed PLAP-stained CST fibres in the MHB region of adult *Sema6A *mutant brains (data not shown). Third, *PlxnA4 *mutant mice show similar defects as *Sema6A *mutants in the caudal medulla, but no defects at the MHB and no hypoplasia of the CST. The later defect within the caudal medulla, which is shared between *Sema6A *and *PlxnA4 *mutants, is thus unlikely to contribute to the observed hypoplasia in *Sema6A *mutants. In fact, many axons that adopt a ventrolateral route within the hindbrain and spinal cord in *Sema6A *and *PlxnA4 *mutants persist into adulthood, and have in *Sema6A *mutants been independently shown to contact appropriate targets in the spinal cord [56].

### Guidance within the medulla

**Figure 10:**
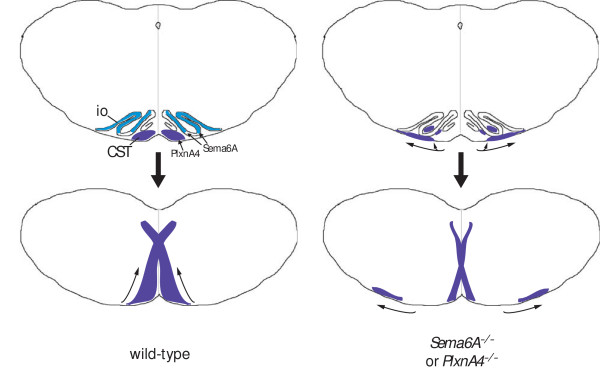
A model for corticospinal tract (CST) guidance at the inferior olive (io) and decussation. CST axons (blue) express PlxnA4. At the level of the olive they are constrained medially in wild-type animals by the expression of Sema6A (cyan, top left). (Note that the olives also express PlxnA4.) Further caudally, this allows the CST axons to respond to cues for decussation (bottom left). In *Sema6A *or *PlxnA4 *mutants (right) the CST axons are not constrained by the inferior olives and a proportion of axons adopt a more lateral position or extend within the olives themselves (top right). At the level of the decussation (bottom right) many CST axons are too far away from the midline to respond to decussation cues and continue to extend in a ventrolateral position. Those that remain near the midline may decussate normally.

Of the axons that make it to the pyramidal decussation point in *Sema6A *mutants, some decussate normally and join the dorsal funiculus, while many take up an abnormal ventrolateral position and continue to project caudally on the ipislateral side. The relative proportions of axons that show this phenotype is somewhat variable, in both *Sema6A *and *PlxnA4 *mutants. Whether this reflects stochastic variation or some molecular distinction between subtypes of axons is not clear. The CST is thought to be pioneered by a small number of axons, with the majority of axons following these, possibly responding more to fasciculation cues on the surface of these axons than to guidance cues in the environment. Small variations in the number of pioneer axons adopting either of the alternative routes might thus be passively transmitted to the follower axons [65]. While the phenotype is most apparent at the decussation itself, in fact the CST axons begin to spread laterally before this point, that is, at the level of the inferior olive. Sema6A is no longer detected on CST axons at this stage but is strongly expressed in the inferior olives, while PlxnA4 is expressed by the CST neurons. This suggests that Sema6A may normally act as a repulsive cue in the olives to constrain CST axons near the midline (Figure 10). Failure to turn dorsally and cross the midline at the normal decussation point may thus be indirect and simply reflect the fact that these axons are no longer situated in a position to respond to the normal medial cues that promote decussation.

A very similar phenotype has been reported in a number of mutants affecting Netrin signalling [20]. In *Unc5c *(formerly *Unc5h3*) mutants, a reduced decussation is observed, with a substantial proportion of axons projecting ventrolaterally. This is preceded in the hindbrain by a lateral broadening of the descending CST, rostral to the level of the decussation. The CST eventually splits into two distinct domains, one close to the midline, which decussates, and one more lateral, which continues to project ipsilaterally. Mutants in *netrin-1 *and a viable mutant of *DCC *show similar phenotypes. These were interpreted as evidence for a direct role of Netrin-1, which is expressed at the midline in the region of the decussation, as a guidance factor for CST axons at this choice point [20]. However, the involvement of Unc5c, which normally mediates repulsion from sources of Netrin-1, complicates this interpretation. Our results suggest an alternative model, which is that the decussation defect in CST guidance is secondary to the profound defect in the migration of inferior olivary neurons observed in these mutants [66]. This would be consistent with the lateral broadening of the CST prior to the decussation point in these mutants. Our data suggest that the CST is normally actively hemmed in by the inferior olive, molecularly mediated by Sema6A (Figure 10). If this structure is not formed properly, or if it lacks Sema6A expression, then CST axons will spread laterally and will not decussate properly. (Notably, the olive forms normally in *Sema6A *mutants.) Interestingly, defects in the formation of the inferior olivary nuclei have also been noted in Joubert syndrome, where pyramidal decussation of the CST also fails to occur [27,29].

A similar phenotype is observed in *NCAM *mutants, where a proportion of CST axons adopts a ventrolateral position, while the rest project dorsally and either cross the midline normally or join the ipsilateral dorsal funiculus [19]. In this case, the laterally misrouted axons are detectable at early postnatal stages but do not persist in adults. The cellular basis of this phenotype is not entirely clear; it could involve a disruption of the homophilic fasciculation of follower axons on pioneers or the involvement of NCAM in mediating responses to other guidance cues [67]. In addition, the integrity of the olive in *NCAM *mutants has not been reported.

### Role of plexins

Sema6A has two known binding partners, PlxnA2 and PlxnA4, which have been shown to act as receptors for Sema6A or as antagonists to Sema6A in different contexts [40,42,45,52]. Our expression analyses show that both plexins are expressed in deep layer V of primary motor cortex, the origin of CST axons. Sema6A is expressed on CST axons at early stages but is not detected at perinatal stages, when CST axons are navigating through the hindbrain. At these stages it is expressed more selectively in upper layer V of M1. Analyses of *PlxnA2 *and *PlxnA4 *mutants suggest that PlxnA4 is the important player in the guidance of CST axons, while PlxnA2 has no essential function. *PlxnA4 *mutants show a qualitatively identical phenotype to *Sema6A *mutants at the level of the pyramidal decussation, although more axons reach that point in *PlxnA4 *mutants as they do not show any defects at the MHB. An abolished interaction of PlxnA4 and Sema6A is thus sufficient to explain the defect at the decussation choice point. The expression patterns of these molecules favour a classic mode of action in this context; that is, Sema6A acts as a guidance ligand for the receptor PlxnA4. Interestingly, an overlapping role for PlxnA3 in this process has also been demonstrated [56]. These authors observed the presence of ventrolaterally projecting CST axons at low penetrance in either *PlxnA3 *or *PlxnA4 *single mutants but at very high penetrance in double mutants. However, PlxnA3 has not been shown to bind Sema6A directly.

Plexins are also well known co-receptors for secreted semaphorins, which have been indirectly implicated in CST axon guidance. As mentioned above, ablation of *L1 *in mice results in a failure of CST axons to decussate at the caudal medulla and, after correct contralateral or aberrant ipsilateral entry into the dorsal funiculus, to extend down the spinal cord beyond cervical levels [12]. In cortical slice cultures, wild-type but not L1-deficient cortical axons are repelled by Sema3A secreted from the ventral spinal cord and this repulsion requires formation of a co-receptor of L1 and neuropillin-1 (*in cis*) [61,68]. In line with this idea, mice expressing L1 with a deleted homophilic binding site that leaves binding to neuropilin-1 intact show a normal CST [69]. However, direct involvement of Sema3A in CST guidance has not been demonstrated. In fact, recent analyses of *Sema3A *mutant mice did not reveal any CST guidance defects [70], indicating the involvement of other molecules in L1-mediated CST guidance at the pyramidal decussation. A lack of defects in *neuropilin-1 *mutant mice [56] also suggests that transmembrane, rather than secreted semaphorins, may be the major players *in vivo *in CST guidance and that the phenotypes of *Plxn *mutants at this choice point reflect their role in direct signalling from transmembrane semaphorins.

The fact that neither the *PlxnA2 *nor the *PlxnA4 *single mutants have a CST defect at the MHB suggests either that Sema6A interacts with some other molecule at this choice point or that PlxnA4 and PlxnA2 compensate for each other. Analyses of double mutants of *PlxnA2 *and *PlxnA4 *did not reveal any additional defects at the MHB, however. *PlxnA3;PlxnA4 *double mutants also do not show any defect at the MHB [56]. Though we can not rule out the possible involvement of PlxnA1 at this point [43], or of additional redundancy between plexins, it seems most likely that the function of Sema6A at this choice point, whether due to expression on the CST axons and/or in the surrounding region, is either homophilic or involves an as yet unknown interacting partner.

## Conclusion

Our findings identify Sema6A as a major contributor to the guidance of CST axons at multiple choice points. They highlight the active nature of guidance at the MHB and pons and the possible importance of the inferior olive as a source of guidance information in the hindbrain. PlxnA4 is implicated as a receptor for Sema6A in the hindbrain but the functions of Sema6A at the MHB appear independent of its known interactors, and may be mediated by Sema6A expression in CST axons themselves at this stage. Finally, the involvement of Sema6A in the guidance of CST axons during development has implications for therapeutic approaches to stimulate regeneration of spinal nerves. In particular, the strong expression of Sema6A by oligodendrocytes may contribute substantially to the growth-inhibitory environment of the adult spinal cord [33,71].

## Materials and methods

### Animals and histology

*Sema6A *mutant mice were described previously [18,53]. Genotyping of *Sema6A *mice was carried out as described [44]. *PlxnA2 *and *PlxnA4 *mutant mice were described in [45] and [40], respectively. Staining for expression of the reporter gene PLAP was carried out on 100 μm vibratome sections of adult brains as described [18].

### DiI tracing of the CST

To identify guidance defect of CST axons during development, the tract was traced with the lipophilic fluorescent dye 1,1'-dioctadecyl-3,3,3',3'-tetramethylindocarbocyanine perchlorate (DiI). After sacrificing mice at P4, the skullcap was removed, leaving the brain in the base of the skull, and three DiI crystals were placed unilaterally into the motor cortex (Figure 3c'). The brains were left in phosphate-buffered saline for 3 h and subsequently immersion fixed and kept in phosphate-buffered saline containing 4% paraformaldehyde. After 5 to 7 months, the brains were removed and frontal vibratome sections, 50 μm in thickness, were prepared.

### Immunohistochemistry

To visualize the CST at P10, immunostaining was carried out on 40 μm vibratome sections using monoclonal rat anti-L1 (1:200; Chemicon, now Millipore, Billerica, MA, USA) or mouse anti L1 (1:500; Abcam, Cambridge, UK) antibodies as described previously [13]. The blocking solution contained 1% bovine serum albumin, 5% normal serum, and 0.5% Triton-100. Primary antibody was visualized with Cy2- or Cy3-conjugated anti-rat or -mouse IgG (Jackson ImmunoResearch, West Grove, PA, USA). For protein expression analysis at E17.5 and P1, we used goat anti mouse Sema6A (1:100; R&D Systems, Abingdon, UK) together with mouse anti-neurofilament-M (clone 2H3, 1:50; DSHB, University of Iowa, Iowa City, USA) as a reference staining of fibres using the same protocol except for an initial 30 minute incubation of sections in 20 mM sodium citrate, pH8.5 at 80°C for antigen retrieval.

### RNA *in situ *hybridization

*In situ *hybridization was performed on free-floating 50 μm vibratome sections of P1 and P4 brains, as previously described [72]. The following digoxigenin-labeled RNA probes were used: Sema6A (kind gift of W Snider), PlxnA2 and PlxnA4 (from the lab of M Tessier-Lavigne).

## Abbreviations

CST: corticospinal tract; DiI: 1,1'-dioctadecyl-3,3,3',3'-tetramethylindocarbocyanine perchlorate; E: embryonic day; M1: primary motor cortex; MHB: mid-hindbrain boundary; P: postnatal day; PLAP: placental alkaline phosphatase; S1: primary somatosensory cortex.

## Competing interests

The authors declare that they have no competing interests.

## Authors' contributions

AER designed and performed experiments, analysed data, and wrote the paper. GEL designed and performed experiments, and analysed data. FS provided reagents and samples. HF provided reagents and samples. KJM designed experiments, analysed data, and wrote the paper.

## Supplementary Material

Additional file 1 Specificity of the goat anti-mouse Sema6A antibody. Sema6A immunohistochemistry on E17.5 coronal (a, b, d-g) and P0 sagittal (c) brain sections of Sema6A homozygous mutant (S6A^-/-^) (a, d, f) and wild-type (wt) (b, c, e, g) mice. The Sema6A antibody binds specifically to the deeper part of the external granule cell layer (EGL in (c), asterisk in (b)) of the cerebellum of E17.5 (b) and P0 (c) wild-type mice, as described previously [44], but not to the EGL in Sema6A mutants (a) (asterisk). In these mutants, the antibody detects the mutated Sema6A protein [18,65], which is located only in cell bodies in all brain regions investigated, such as granule cells of the cerebellum (a) or in cells that form the inferior olive (IO) of the medulla (d). Note that in the mutants, the corticospinal tract (CST) (f) is not stained, which is in contrast to that of wild type (g). IGL, internal granule cell layer. Scale bars 100 μm: the scale bar in (b) is for (a, b); the scale bar in (g) is for (c-g). Click here for file

Additional file 2 Expression of PlxnA1 and PlxnA3 in motor cortex. *In situ *hybridization on coronal brain sections of P4 wild-type mice at low (a, c) and higher (b, d) magnification. Within the primary motor cortex (M1), PlxnA1 is only moderately expressed throughout layers, but lowest in layers V and VI. In this area PlxnA3 is expressed at moderate to strong levels in upper layers, but only moderately in lower layers. Scale bar in (c) is 200 μm and is for (a, c); scale bar in (d) is 100 μm and is for (b, d). Click here for file
