# Supplementary figures and images for: Semaphorin-6A controls guidance of corticospinal tract axons at multiple choice points
Source: Neural Dev. 2008 Dec 8;3:34. doi: 10.1186/1749-8104-3-34 (PMC2647909; doi:10.1186/1749-8104-3-34)

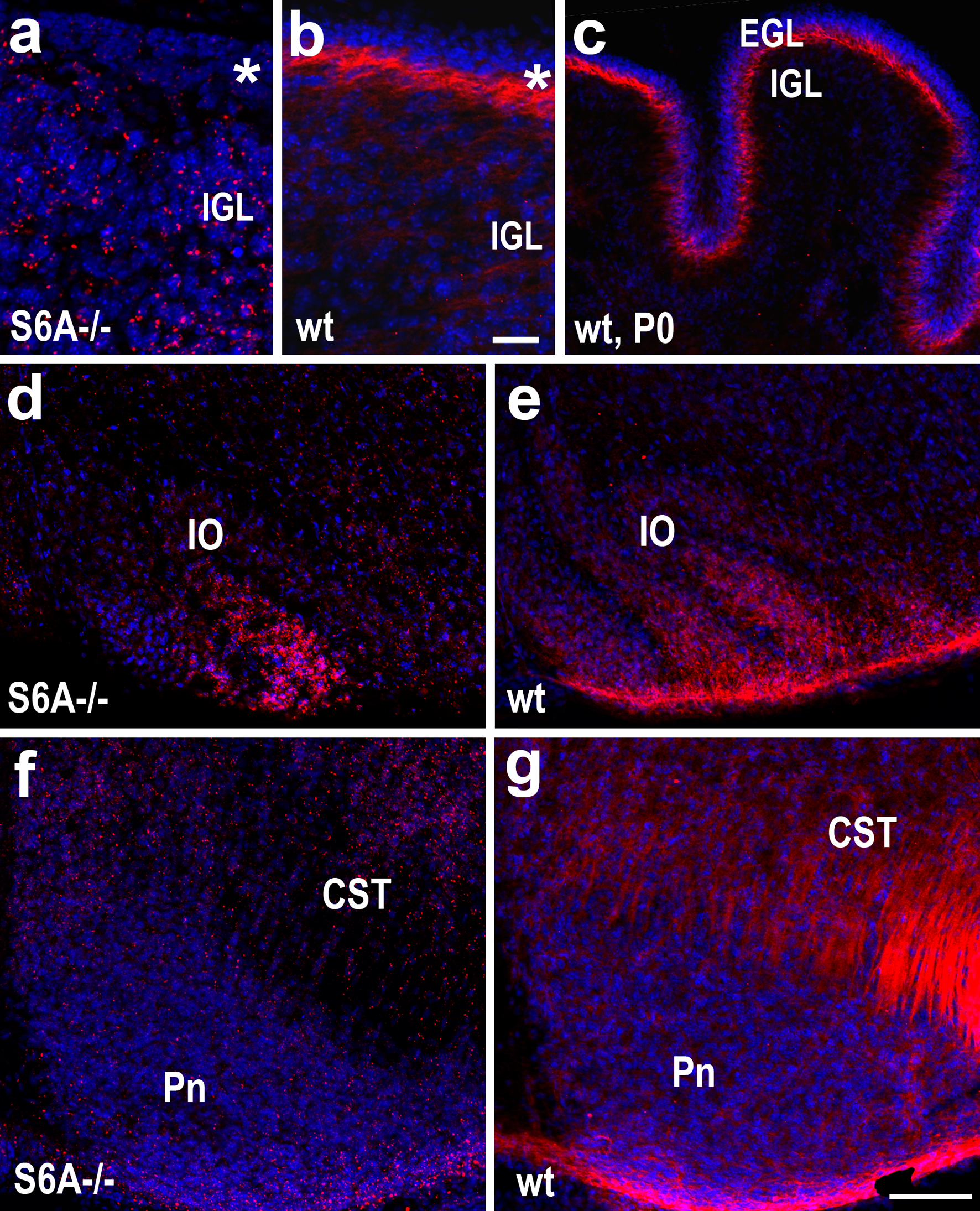

Supplement: Additional file 1 — Specificity of the goat anti-mouse Sema6A antibody. Sema6A immunohistochemistry on E17.5 coronal (a, b, d-g) and P0 sagittal (c) brain sections of Sema6A homozygous mutant (S6A-/-) (a, d, f) and wild-type (wt) (b, c, e, g) mice. The Sema6A antibody binds specifically to the deeper part of the external granule cell layer (EGL in (c), asterisk in (b)) of the cerebellum of E17.5 (b) and P0 (c) wild-type mice, as described previously [44], but not to the EGL in Sema6A mutants (a) (asterisk). In these mutants, the antibody detects the mutated Sema6A protein [18,65], which is located only in cell bodies in all brain regions investigated, such as granule cells of the cerebellum (a) or in cells that form the inferior olive (IO) of the medulla (d). Note that in the mutants, the corticospinal tract (CST) (f) is not stained, which is in contrast to that of wild type (g). IGL, internal granule cell layer. Scale bars 100 μm: the scale bar in (b) is for (a, b); the scale bar in (g) is for (c-g). [file 1749-8104-3-34-S1.tiff]

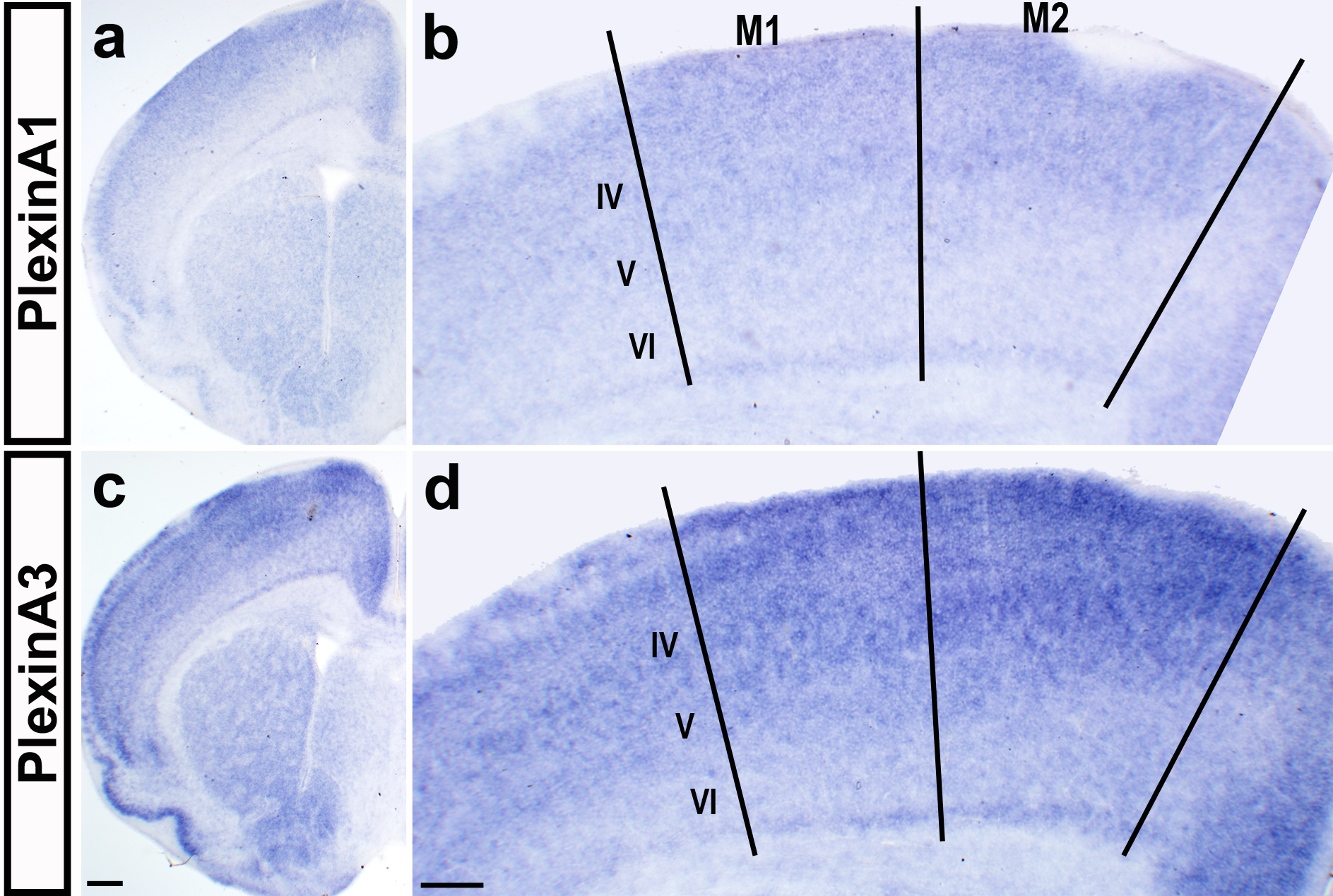

Supplement: Additional file 2 — Expression of PlxnA1 and PlxnA3 in motor cortex. In situ hybridization on coronal brain sections of P4 wild-type mice at low (a, c) and higher (b, d) magnification. Within the primary motor cortex (M1), PlxnA1 is only moderately expressed throughout layers, but lowest in layers V and VI. In this area PlxnA3 is expressed at moderate to strong levels in upper layers, but only moderately in lower layers. Scale bar in (c) is 200 μm and is for (a, c); scale bar in (d) is 100 μm and is for (b, d). [file 1749-8104-3-34-S2.tiff]
